# Human Hair Keratin Composite Scaffold: Characterisation and Biocompatibility Study on NIH 3T3 Fibroblast Cells

**DOI:** 10.3390/ph14080781

**Published:** 2021-08-09

**Authors:** Jamal Moideen Muthu Mohamed, Ali Alqahtani, Adel Al Fatease, Taha Alqahtani, Barkat Ali Khan, B. Ashmitha, R. Vijaya

**Affiliations:** 1 Department of Pharmaceutical Technology, BIT Campus, Anna University, Tiruchirappalli 620024, India; jmuthumohamed@gmail.com (J.M.M.M.); scisp2@aliyun.com (B.A.); 2 Department of Pharmacology, College of Pharmacy, King Khalid University, Guraiger, Abha 62529, Saudi Arabia; amsfr@kku.edu.sa (A.A.); ttaha@kku.edu.sa (T.A.); 3 Department of Pharmaceutics, College of Pharmacy, King Khalid University, Guraiger, Abha 62529, Saudi Arabia; afatease@kku.edu.sa; 4 Faculty of Pharmacy, Gomal University, Dera Ismail Khan 29050, Pakistan; barkat.khan@gu.edu.pk

**Keywords:** composite scaffold, human hair keratin, gentamicin sulphate, polyvinyl alcohol, NIH 3T3 cells, MTT assay

## Abstract

The aim of this study was to transform human hair keratin waste into a scaffold for soft tissue engineering to heal wounds. The keratin was extracted using the Shindai method. Keratin and polyvinyl alcohol (PVA) was cross-linked with alginate dialdehyde and converted into a scaffold by the freeze-drying method using gentamycin sulphate (GS) as a model drug. The scaffold was subjected to Fourier transform infrared spectra (FTIR), swelling index, porosity, water absorption, scanning electron microscopy (SEM), differential scanning calorimetry (DSC), thermal gravimetric analysis (TGA), X-ray diffraction (XRD), drug release, and cell viability (MTT) analysis. The scaffold was tested for keratinocyte growth using the murine fibroblast cell line (NIH 3T3 cells). The outcome from the keratin had a molecular weight band between 52–38 kDa in SDS-PAGE (Sodium dodecylsulfate-Polyacrylamide gel electrophoresis). A porous scaffold was capable of water absorption (73.64 ± 14.29%), swelling ability (68.93 ± 1.33%), and the release of GS shown as 97.45 ± 4.57 and 93.86 ± 5.22 of 1:4 and 1:3 scaffolds at 16 h. The physicochemical evaluation revealed that the prepared scaffold exhibits the proper structural integrity: partially crystalline with a strong thermal property. The scaffold demonstrated better cell viability against the murine fibroblast cell line (NIH 3T3 cells). In conclusion, we found that the prepared composite scaffold (1:4) can be used for wound healing applications.

## 1. Introduction

The process of wound healing lasts for several weeks and involves a number of stages. There have been several attempts in the research with respect to formulation design to heal different kinds of wounds [1,2]. Among these, scaffolds have been particularly suitable for topical wound healing due to the flexibility in their design, especially when used in the soft tissue engineering field. The scaffolds could be composed of hybrid materials based on the therapeutic requirements; hence, we focused on a wound that occurs on the skin [3]. The present research on keratin, a major filament protein found in human hair, was selected; as it has been successfully employed in recent research as a biomaterial for nerve regeneration scaffolds in tissue engineering application of and hemostasis [4].

Human hair keratin is analogous to the extracellular environment in the body. However, keratin alone is not sufficient to provide mechanical integrity and flexibility, and thus materials such as polyvinyl alcohol (PVA), gelatin, and chitosan have been combined with keratin to improve the integrity of the scaffold [5,6]. The blending of keratin with a suitable synthetic polymer (PVA) is essential to create a scaffold with ideal mechanical properties [7,8]. Specifically, PVA has been found to have an effective film-making property when combined with wool keratin. However, since wool keratin is obtained from animal origins, it may lead to the possibility of immunogenicity. PVA has high hydrophilicity, chemical, and mechanical resistance [9]. Therefore a polymer that is a combination of both synthetic and biological materials can be used to develop a scaffold with improved biological functionality and mechanical properties with controlled degradation and biocompatibility [10]. The presence of Arginine-Glycine-Aspartic Acid (RGD) and LVD cell-binding motifs in keratin, and the adhesive nature of the PVA materials blended is more suitable for the preparation of scaffold tissue engineering application [11]. Here, incorporated antibiotic gentamycin sulphate (GS) could support the quick healing of wound by preventing the infection. GS is hygroscopic and very stable in acidic and alkaline conditions. It is not absorbed orally and causes adverse effects upon parenteral administration with a short elimination half-life of 2 h. Hence, the controlled release of GS may help enhance the wound healing rate by increasing the drug concentration at the wound site.

Epithelial cell keratins make up the type I (K9–K20) and type II (K1–K8) intermediate filament proteins. In glandular epithelia, K8 becomes phosphorylated on S73 (71LLpSPL) in human cultured cells and tissues during stress, apoptosis, and mitosis. Of all known proteins, the context of the K8 S73 motif (LLS/TPL) is unique to type II keratins and is conserved in epidermal K5/K6, oesophagal K4, and type II hair keratins, except that serine is replaced by threonine. In conclusion, type II keratins of proliferating epithelia undergo phosphorylation at a unique and conserved motif as part of physiological mitotic and stress-related signals [12].

The controlled release of antibiotics, such as clindamycin and mupirocin, from scaffolds into the body have been found to support the topical wound healing process. Further, the scaffold possesses a porous architecture that can restore, maintain, or improve tissue function [13]. This porous architecture is essential for the wound healing process in that it allows for the diffusion of nutrients and metabolites for cell growth. A cross-linking agent alginate dialdehyde (ADA) was added to the scaffold in our study to achieve controlled drug release and enhance the moisture sensitivity, thermal stability, and mechanical properties of the keratin-based scaffold [14].

The present study mainly emphasis is on the preparation of PVA/keratin mixture scaffolds. PVA was chosen for this purpose due to its extraordinary biocompatibility, high water absorption capacity, elasticity, and mechanical mobility and storability. Keratin was selected due to its biocompatibility and biodegradability, integrin-binding fields and the RGD motif, which enables cell attachment. Keratin develops the activity and gene expression of Schwann cells by a chemotactic mechanism, which increases their attachment and proliferation, and up-regulates the expression of important genes, which considerably improves electrophysiological activities [15].

The novelty of this work was that the composite scaffold was prepared using human hair waste for soft tissue engineering (wound healing) and evaluated for Fourier transform infrared spectra (FTIR), swelling, water absorption, porosity, surface morphology, thermal analysis, polymorphic changes, in vitro drug release, and the MTT assay on the murine fibroblast cell line (NIH 3T3 cells).

## 2. Results and Discussion

The Shindai method was selected for the extraction of human hair keratin, as this method yields α- keratin and does not use any detergents [16]. The scaffold that was developed using a 1:3 and 1:4 ratio of keratin and PVA formed a continuous porous composite scaffold. A 2% (*w/w*) concentration of PVA with a 0.5% of 0.75 mL alginate dialdehyde cross-linking agent as reported by Santos and co-workers prepared an optimised scaffold [17]. Another study performed by Xu and his team reported that the lower keratin concentration yielded scaffolds with high porosity [18]. Hence, the ratio of keratin was kept to a minimum while the amount of PVA was increased in the process.

### 2.1. Quantification of Extracted Keratin by SDS-PAGE

**Figure 1:**
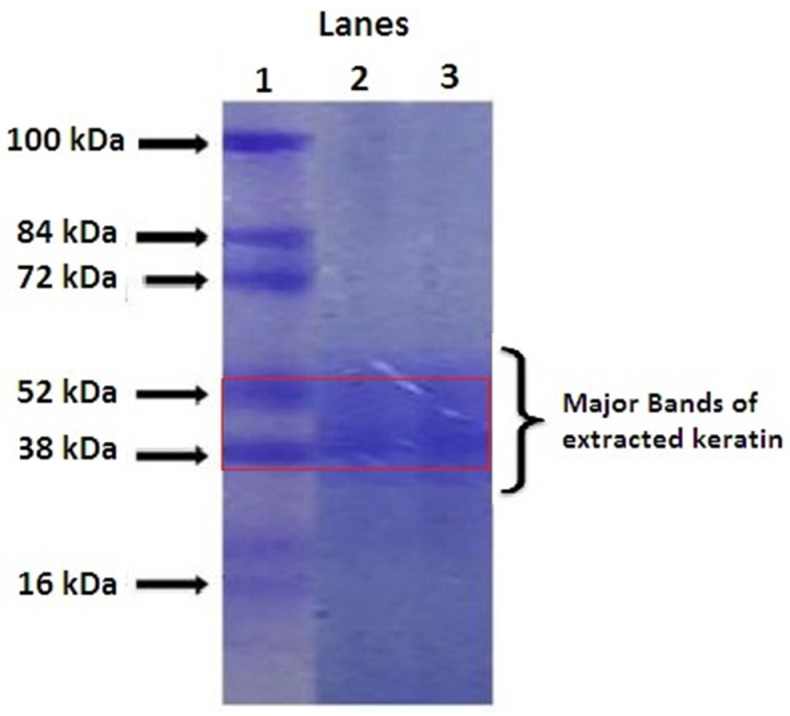
SDS PAGE analysis of keratin (Lane 1: Serum, Lane 2: Protein marker, Lane 3: Extracted keratin).

The SDS-PAGE method was used to ensure the presence of keratin, as the mixture could contain both keratins and matrix proteins. Prior to performing the electrophoresis analysis, the extracted keratin solution was subjected to dialysis and held at −80 °C for 2 days to prevent any accumulation. The extracted keratin showed a major band at the molecular weight of 52–38 kDa (Lane 3), which represents the low sulphur-containing “alpha” keratin of hair (Figure 1) as similar to the previously reported studies [19].

These bands match with the protein marker (Lane 2), and multiple bands have been observed in the serum (Lane 1). These α keratins can assemble together to form a microfibrous structure known as keratin and helps the suitable scaffold development. The extracted keratin was quantified using the Bradford colourimetric method as reported by Arsalan and co-workers [20].

### 2.2. FTIR

**Figure 2:**
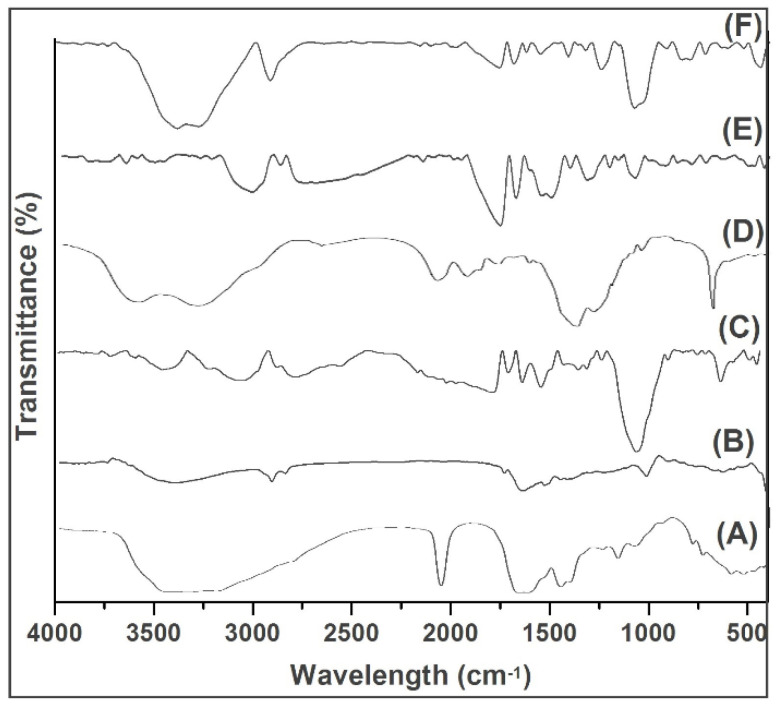
FTIR spectra of (**A**) Reference keratin, (**B**) Extracted keratin, (**C**) PVA, (**D**) GS, (**E**) scaffold (1:3), and (**F**) scaffold (1:4).

Generally, the FTIR analysis was based on the identification of absorption bands concerned with the vibrations of functional groups present in the molecules. The intermolecular interaction of the substances blend together to prepare the scaffold to be studied, and this, their compatibility can be determined. From the FTIR spectra of reference keratin (Figure 2A), the absorption peaks at 1649, 1540, and 1235 cm^−1^ were the typical peaks of amide I, II, and III, respectively [21]; the peaks at 3562 cm^−1^ were the typical intense bond of the N–H stretching vibration, and the strong peak at 1028 cm^−1^ reveals the S–O stretching vibration (symmetric) of cysteine-S-sulfonate residues. The spectrum pattern of the extracted keratin (Figure 2B) revealed a band at 3422.06 cm^−1^ due to N-H stretching vibrations, and the slight increase in vibration might be attributed to the impact of water molecules. The 1642.84 cm^−1^ band indicates C=O stretching of amide I, representing alpha helix and beta structure in random conformation. A band at 1460.81 cm^−1^ shows N-H bending vibrations of amide II of keratin. The obtained keratin possesses functional bands that correspond to those of the reference keratin, as evidenced from its FTIR spectral analysis (Figure 2A) as reported by Selmin and colleagues [21]. The difference between these keratins, the reference keratin absorption peak for strong Amide A vibrations was observed, contrary to the extracted keratin, absorption peak for weak Amide II, III were obtained from the primary N-H stretching vibrations but also is contributed by the Fermi resonance from the first overtone amide II.

An increase in the peak shift at 3739.3 cm^−1^ representing C-H stretching shows the strong association of the C-H units of scaffold components such as keratin, PVA, and GS (Figure 2). The appearance of bands at 1764.55 and 1692.23 cm^−1^ are indications of carboxyl groups, such as amide or carboxylate, which might be due to the conjugation of OH and amine group presents in the scaffold components (Figure 2E). A sharp and altered small peak at 1627.63 cm^−1^, between 1615–1495 and at 612.39 cm^−1^ represents a double bond and the overlapping of functional groups of aliphatic and aromatic compounds. A strong band occurred at 2919.7 cm^−1^ (Figure 2F). Overall, the strong amide band was observed with the scaffold prepared with a 1:4 ratio exhibited as a result of sulphides of keratin molecules.

Thus, the FTIR analysis revealed the compatibility between the components of the scaffold with mere association and/or conjugation of their groups. The studied effect might be possible to connect all the components together in order to form a continuous scaffold.

### 2.3. DSC

**Figure 3:**
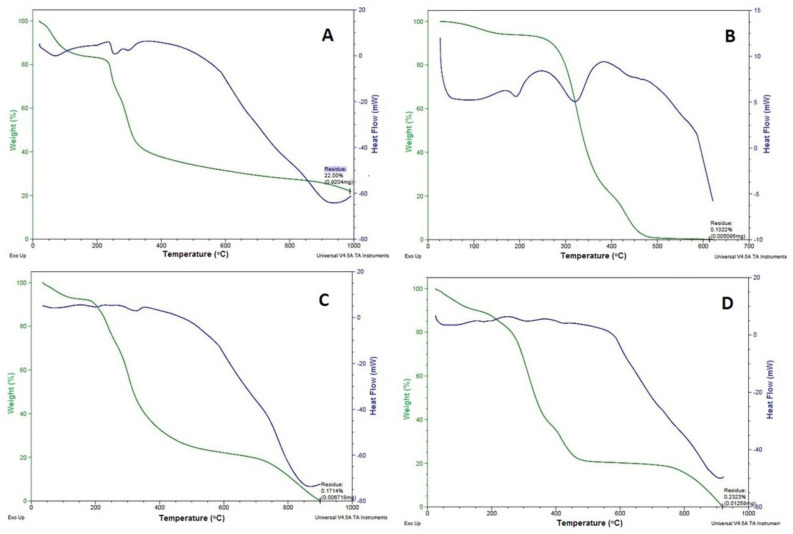
DSC (blue) -TGA (green) curves of (**A**) Keratin, (**B**) PVA, (**C**) GS, and (**D**) 1:4 composite scaffold.

The transitions that take place in the samples (Figure 3; blue colour curve), when subjected to heat treatment reflect the thermal behaviour of protein-based composite scaffolds. The endothermic peaks at 100–120, 190, and 250–400 °C represent the transitions of hydrogen bound water, the α-helix denaturation of keratin (Figure 3A), and the crystalline temperatures of the PVA (Figure 3B) and the GS (Figure 3C), respectively. At a PVA content of 20% in the mixture of the scaffold, the ∆He value for the vaporisation of bound water increased from 190.0 to 232.7 J/K, signifying a higher aqueous binding capacity of the mixed scaffold, this effect due to PVA’s higher hydrophilicity. The addition of alginate dialdehyde caused a reduction of the ∆He value to about 132.7 J/K due to the chemical cross-linking effect. This evidence of the keratin/PVA scaffold with alginate dialdehyde-induced cross-linking leads to less hydrophilic, as reported by Dou et al. [22]. However, the mixture of the scaffold has no noticeable effect on the transition temperature (Tm) of PVA and keratin; on the contrary, it was reduced significantly. The decrease in the Tm value of keratin inside the keratin/PVA composite relative to the pure keratin may be due to a reduction in the crystalline/amorphous regions ratio during the dissolution process. Crosslinking of keratin/PVA composite with alginate dialdehyde ensued in back increase in the Tm of keratin to 248 °C, prospectively due to crystallisation of part of the amorphous phase of previously dissolved keratin into crystalline form. This suggests the thermal stability of composite materials in the scaffold without having any additional, or new thermal transitions complied with the reported by Kakkar et al. [23]. However, a slight (increase) shift in the transition temperature peak was observed in the 1:4 composite scaffold (Figure 3D) when compared with DSC thermograms of keratin, PVA, and GS. This may be advantageous in terms of increased thermostability of the materials when developed into a scaffold. It may be the case that a strong affinity existed between the composite materials, which thus made them more stable at higher temperatures.

### 2.4. TGA

The TGA was carried out to quantify the thermal degradation of the scaffold in a controlled manner. TGA reveals the total degradation of materials at a particular temperature (Figure 3; green colour curve). A weight loss between 200 and 400 °C incorporates the typical curve of keratin (60% weight loss between 225.09 and 387.87 °C; Figure 3A), PVA (54% weight loss between 322.24 and 323.87 °C; Figure 3B) and GS (54% weight loss between 282.24 and 284.87 °C; Figure 3C). However, the moisture loss for these materials was found to be in the range between 100 and 150 °C for keratin and PVA and 100–120 °C for GS [24]. In the case of the composite scaffold, an initial weight loss occurred at 59–110 °C due to the loss of moisture, and a 54% weight loss occurred between 265.24 and 277.87 °C, and 100% weight loss occurred between 200 and 400 °C (Figure 3D). The weight loss due to moisture occurred at a very low temperature (59–110 °C) for the scaffold compared with its component materials (100–150 °C). Hence, the greater values of weight loss in the first degradation phase indicate the existence of a chemical decomposition progression revealed from bond scission (carbon-carbon bonds). The lower weight loss may indicate the breaking of the ester linkages and the second stage to the degradation of the whole polymer. This might be due to the enhanced water absorption and water loss capacity of the porous scaffold formed using the freeze-drying technique as studied by Barua and co-workers [25].

### 2.5. XRD

**Figure 4:**
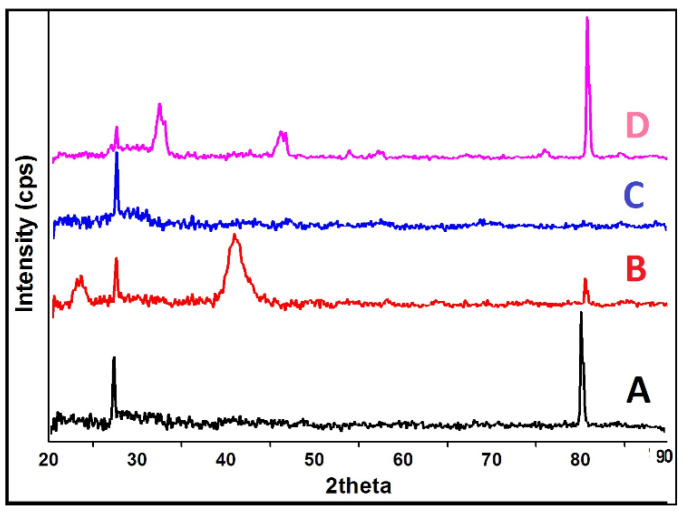
XRD analysis of (**A**) Keratin, (**B**) PVA, (**C**) GS, and (**D**) 1:4 composite Scaffold.

The X-ray diffractograms of the composite materials and the scaffold (Figure 4D) are shown on the phase change effects that occurred during scaffold preparation and further freeze-drying processes. The Amorphous (keratin) and semi-crystalline (PVA) nature with the absorption peak was found at 20, 21, 23, as shown in Figure 4A,B. In Keratin’s case, there were no phase changes observed except for an increase in peak intensity at 80°. The presence of semi-crystalline PVA in the scaffold may be due to the addition of a dialdehyde starch or water cross-linking agent. This kind of partially crystalline behaviour may help in maintaining the stability of the product during storage, as reported by Mabrouk and co-workers [26]. A broad peak at 31–33° with increased intensity reflects the partially crystalline nature of GS (Figure 4C), which might be due to the presence of glycoside molecules, which have a strong tendency to crystallise at lower freezing temperatures. While keratin and PVA interacted with GS, the corresponding peak of GS at 33° disappeared (Figure 4D). This might be due to molecular transition into its amorphous form. This may be highly effective for promoting a slow degradation of biomaterial at the wound site, allowing for a longer duration of action for better and more complex wound healing processes (i.e.,) the biomaterial could coincide with the chronological (time) pattern of the wound healing process. Overall, the XRD revealed the crystalline nature of the scaffold partially converted into amorphous.

### 2.6. Swelling, Water Absorption and Porosity

The application of the swelling index is important in the case of physicochemical characteristics of the scaffolds. Swelling behaviour is an intrinsic property of a scaffold, where the scaffold swells, which might be the penetration of solvent into the void space among the polymeric arrangement of the networks. In some cases, swelling behaviour can also be influenced by external triggers such as ionic strength, pH, and the temperature of the environment. This study could be performed after PVA has been cross-linked of the prepared scaffolds. Principally, the pore diameter of the scaffold increases is directly linked with the swelling behaviour such as size (1.5–2%), water absorption (72–83%) and weight (25–30%) of the scaffold composite shown in Table 1. The results proposed that the increase in the amount of PVA leads to decreases in the swelling behaviour and increases the water absorption of the scaffold. The effect could not affect the dissolution with no significant difference regardless of keratin/PVA content due to hydrophilicity. The swelling behaviour of keratin/PVA scaffolds (1:3 and 1:4) with respect to time. The significant differences in swelling percentage were observed between the 1:3 and 1:4 scaffold in pH 7.4 medium. This might be due to the formation of more polymeric networks with the increased concentration of PVA. Choudhury et al. studied the penetration of solvent into the porous scaffold, and its diffusion through the polymer matrix causes local relaxation of polymer segments leads to swelling [27].

The percentage of water uptake (Table 1) for the 1:4 scaffold was found to be 83.64 ± 14.29% in water after 24 h and was not significantly different from the 1:3 scaffold (72.93 ± 9.26%). This water absorption character of scaffold is due to holding water by capillary force in the interconnected pores and that of hydrophilicity of PVA. The mixture of PVA with keratin reported comparatively slow water absorption due to a more rigid network formed by the inter-and intra-polymer reactions and also to the reduction of hydrophilic groups in the PVA/keratin mixture. The latter is also due to the amine groups of keratin being more reactive than to hydroxyl groups of PVA. Generally, this water absorption capacity depends upon the distribution pattern of pores present within the polymeric structure, which further influences the mechanical properties of the scaffold. However, the percentage of water absorption obtained matches that of earlier reports and is suitable for wound healing processes in that it holds cellular infiltrates for growth [28].

The porosity (Table 1) of 68.93 ± 1.33% obtained for the 1:4 composite scaffold was comparable to the 1:3 ratio (66.05 ± 1.97%). Porosity reflects the size of the pores present in the scaffold, and higher porosity can be obtained by increasing the amount of PVA, lesser cross-linking agents and keratin. The scaffold’s swelling properties and pore size would depend on solution ions and other solution-related factors, which would be relevant to the folding or unfolding of the keratin protein with a specific -S-S- content. Previous studies have found that lower keratin concentrations tend to increase the porosity of the scaffold [29]. The formation of a highly porous scaffold has a significant influence on the wound healing process in that it improves the transport of materials in and out of the scaffold.

### 2.7. SEM

**Figure 5:**
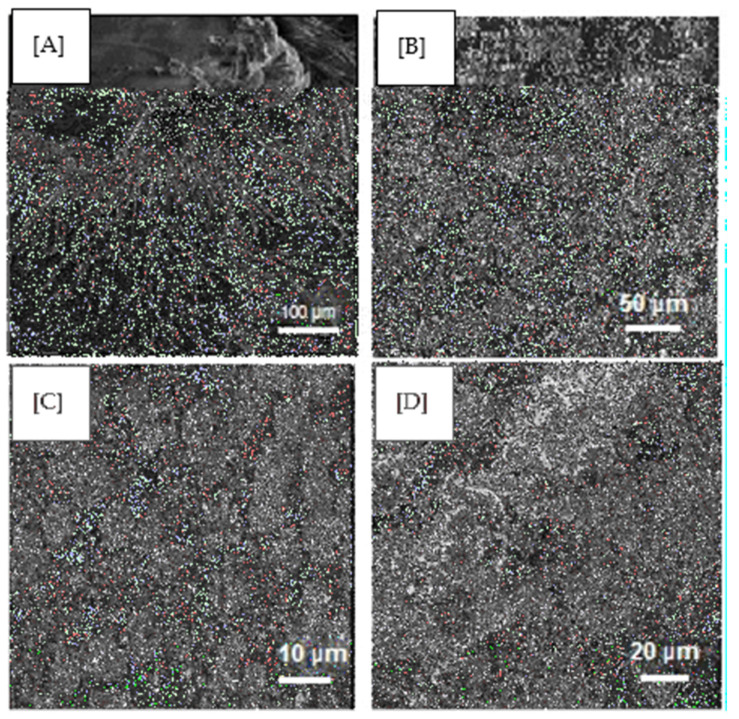
SEM images of (**A**) GS, (**B**) Keratin, (**C**) 1:3 composite scaffold (**D**) 1:4 composite scaffold.

The SEM visualises the heterogeneous porous architecture of scaffolds, as shown in Figure 5, as well as their interconnectivity and fibrous arrangements. The GS (Figure 5A) and extracted keratin (Figure 5B) shows the characteristic crystalline structure with the average particle size of 5 µm and 200 µm, which complied with the reported data [30]. Figure 5C,D represents the surface of the scaffold of 1:3 and 1:4, respectively, having even roughness evident of PVA absolutely cross-linked. This kind of morphology is essential for the proper diffusion of nutrients, metabolites and for the cell population to undergo differentiation and growth in and out of the scaffold, particularly for skin regeneration.

### 2.8. In Vitro Release

**Figure 6:**
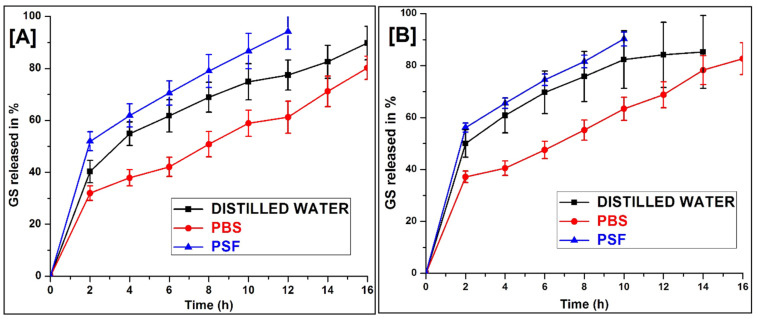
In vitro of GS release from (**A**) 1:3, and (**B**) 1:4 scaffold in different dissolution medium (Mean ± SD, *n* = 3).

The GS release from the composite scaffold in PSF, water and a PBS (pH 7.4) that lasted for up to 16 h is shown in Figure 6. There was an immediate release of GS followed by a slow-release observed with both scaffolds. The GS release was found to be high in the order of PSF > water > PBS. In the case of PSF, approximately 90% and 86% of the GS had been released within 10 h, whereas it took 14 h and 16 h for the water and the PBS scaffold 1:3 (Figure 6A) and 1:4 (Figure 6B), respectively, after which degradation of the scaffold structure occurred. The study revealed that GS release is influenced by the fluid environment present in the dissolution medium. The immediate drug release percentage may help provide a high drug concentration at the wound site, prevent infection, and help speed up the healing process, whereas sustained-release maintains GS at the wound site for a long period [31].

The structural integrity of the scaffold was retained in the entire release medium during the study. This might be due to the cross-linked PVA having a greater break strength. It may be possible to extend the drug release in PSF further using pH-controlled polymers in the scaffold, which could also retain the physical integrity of the scaffold in the prolonged process of wound healing [32].

From the degradation behaviour, it was clearly detected that the degradation rate of the 1:3 scaffold was much slower than the 1:4 scaffold. This is due to a higher density of chemical cross-linking between alginate dialdehyde and amine groups of keratin which leads to slower depolymerisation. These results show that after mixing with keratin/PVA offers a relatively controlled degradation rate which is essential for the broken nerve that requires time for its complete recovery.

### 2.9. Biocompatibility Testing by MTT Assay

**Figure 7:**
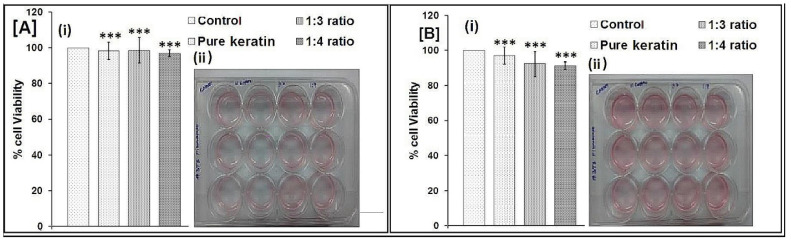
MTT assay of after (**A**) 24 h, and (**B**) 48 h (*** *p* < 0.05 calculated by the One-way ANOVA test).

NIH 3T3 cells were selected for the study because they are easy to preserve and are well known in cell-scaffold interaction studies [33]. The wound healing properties conclude that the keratin and 1:3 and 1:4 scaffold on NTH 3T3 cell lines from MTT-reduction assay of the percentage of viable cells is shown in Figure 7. From the study, the viable cells were rises with the reductions in hours. The behaviour of the composite scaffold on fibroblast cell lines by means of colour formation in terms of optical density at 550 nm is shown in Figure 7. The increase in the intensity of formazan product colour indicates an increase in cell counts in number. An MTT assay showed that there was more than 90% cell viability, as calculated from the OD values obtained after 24 (Figure 7(Aii)) and 48 h (Figure 7(Bii)) of culturing the plates.

The mechanism of wound healing explained by Kelly (2016) found that the keratin activate the keratinocytes and associated proliferations in basement assembly of skin proteins collagen IV and collagen VII, and thus provide the basis for clinically observed efficiency for keratin wound healing applications [34]. The results further show that the structure and composition of the scaffold (1:4) support the fibroblasts greater than the 1:3 ratio. Thus, the scaffold (1:4) was found to be biocompatible with fibroblast cells with a higher percentage of cell viability.

## 3. Materials and Methods

### 3.1. Materials

Hair was collected from a local barbershop, King’s Saloon, Tamil Nadu, India. Polyvinyl alcohol and all other chemicals were purchased from Sigma-Aldrich, Bangalore, India, unless specified otherwise. The mouse NIH-3T3 fibroblast cell lines were purchased from the National Centre for Cell Science (NCCS), Pune, Maharashtra, India. The monolayer of fibroblast cell line NIH 3T3 were developed on and maintained in Dulbecco’s modified Eagle’s medium (sigma) with 10% fetal calf serum (Sigma Aldrich, St. Louis, MO, USA) and supplemented with antibiotics: penicillin (120 units/mL), streptomycin (75 mg/mL), gentamycin (160 mg/mL), and amphotericin B (3 mg/mL) at 37 ºC humidified with 5% CO_2_.

### 3.2. Extraction of Keratin

#### 3.2.1. Preparation of Buffer Solution for Keratin Extraction

The buffer solution was prepared by adding Tris base, 19.79 g of thiourea, 30 g of urea and 5 mL of 2-mercaptoethanol in 100 mL of deionised water. The pH was then adjusted with 8M HCl to 8.5. The Tris HCl was used to keep the keratin extraction buffer solution at a pH of 8.5 (called a Shindai extract). From the prepared extract, thiourea and urea break down the non-covalent bond between the polypeptide chains of amino acids, whereas 2-mercaptoethanol reduces the disulfide bond between amino acids (cysteine) [35].

#### 3.2.2. Extraction of Keratin by Shindai Method

**Figure 8:**
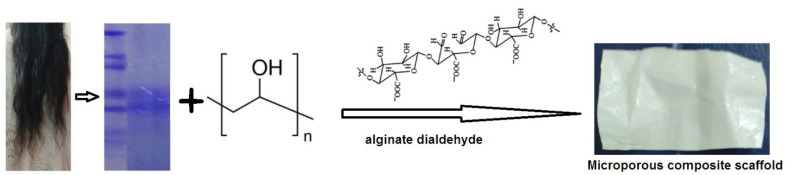
Schematic representation of human hair keratin scaffold fabrication.

Human hair was washed with ethanol and water several times in order to remove any dirt from the surface of the hair. The cleaned hair was put into a 2:1 *v/v* solution of chloroform and methanol for 24 h in order to remove any fat from the surface of the hair. The delipidized hair was washed with distilled water and kept in open air overnight to evaporate the chloroform and methanol [36]. A total of 6 g of delipidized hair was cut into small pieces with an average length of 1 mm, followed by mixing in 100 mL of buffer solution described above and shaken well for 3 min, then kept inside a preheated oven at 500 °C for three days. Following this, the solution was shaken appropriately for 12 h. A 2 µm Whatman filter paper was used to separate the solution containing protein from the cuticle-cortex residue. Then, the filtrate was centrifuged at 15,000 rpm (Remi, 220–240 V, Mumbai) for 20 min at room temperature using a 50 mL Falcon conical centrifuge tube (Fisher Scientific, Chennai, India) to remove small fragments of hair residue. The obtained supernatant was dialysed against deionised water using snakeskin dialysis tubing (Thermo scientific, MW cut-off 3.5 kDa; 16 mm in dm). During dialysis, the water was replaced with deionised water twice over four days [37,38]. Furthermore, the solution started to change to a protein milk-like colour due to aggregation, a polymerisation reaction occurred, and the dialysed protein solution was kept at −80 °C for 48 h. Finally, the frozen protein solution was placed in a lyophiliser (Christ, Alpha1-2 LD plus, Osterode am Harz, Germany) at a pressure of 3.5 Pascal (pa) and temperature at −48 °C for 48 h to fabricate the solid human hair keratin scaffold, as shown in Figure 8.

### 3.3. Preparation of GS Loaded Scaffold

The preparation of the desired pore size of the scaffold, the freezing rate could be controlled by the freeze drying method. The keratin solution (80 µg/mL) and PVA (2% *w/v*) were mixed in the proportions of 1:2, 1:3, and 1:4 and alginate dialdehyde (0.5%) was added drop by drop in each ratio, respectively, with continuous stirring. The gentamicin sulphate (1% *w/v*) was added to the above solution and magnetically stirred (Remi, 2MLH) for 1 h at room temperature (Table 2). The solution was poured into a 3 cm diameter petri dish (glass) lubricated with ethylene glycol as a plasticiser. Finally, the whole mixture was poured into a pre-cooled 24-well plate, frozen at −80 °C overnight and then lyophilised (Christ, Alpha1-2 LD plus, Germany) for two days. The resulting scaffold was washed with phosphate-buffered saline solution (pH 7.4, 0.07 M) at room temperature [39].

### 3.4. Characterisation of Scaffolds

#### 3.4.1. SDS Polyacrylamide Gel Electrophoresis (SDS-PAGE)

Further, the extracted keratin was identified by Sodium Dodecyl Sulphate-Polyacrylamide Gel Electrophoresis (SDS-PAGE) according to the method described by Laemmli [40]. Briefly, the aqueous solution of reduced keratin was subjected to an electrophoresis unit at 15 °C using a 10–15% gradient gel at 250 V and 10 mA. The gel was stained using Coomassie brilliant blue R-250. After the staining and destaining process, the gel was compared for the molecular weight of the sample with a protein marker.

#### 3.4.2. Fourier Transform Infrared (FTIR) Spectral Analysis

The FTIR spectra and structure of extracted keratin and lyophilised composite scaffold was determined and compared using a standard/reference keratin sample obtained using the ATR (Attenuated Total Reflection) technique [41]. The scaffold pellets were prepared by mixing them with potassium bromide for the ATR technique in FTIR spectroscopy studies. Infrared spectra were recorded from 500 to 4000 cm^−1^, using a Jasco FT-IR/6300 spectrophotometer.

#### 3.4.3. Thermal Properties

The thermal characteristics of the scaffold were investigated using a differential scanning calorimeter (DSC) in order to measure their crystallisation temperature (TC) and melting temperature. The analysis was performed at a heating rate of 20 °C/min, from 0 to 1000 °C, under nitrogen gas with a flow rate of 10 mL/min [42]. For the determination of the thermostability of the scaffold, thermogravimetric analysis (TGA) was conducted under 50 mL/min nitrogen gas with a heating rate of 10 °C/min in the range from 0 to 1000 °C by a thermogravimetric curve. The weight changes of the scaffold preparation were analysed by SDT Q600 V20.9 Build 20.

#### 3.4.4. Crystallization Analysis

The crystalline nature of the composite scaffold was determined by X-Ray Diffraction (XRD) patterns performed at room temperature using an analytical X’PERT PRO powder diffractometer with Cu kα radiation operating at a voltage of 40 V [43]. The XRD was taken at 2θ angle range of 5–60°, and the process parameters were: scan step size 0.02 (2θ) and scan step time 0.05 s.

### 3.5. Swelling, Water Absorption and Porosity

The studies were carried out according to the procedure described by Nandha and co-workers [38]. The percentage swelling of the scaffold at equilibrium was calculated by the following equation:

Percentage swelling (%) = [(Ws − Wd)/Wd] × 100

where Wd is the weight of the dry scaffold and Ws is the weight of the swollen scaffold. Similarly, the percentage of water absorbed was calculated by the following equation.

Percentage water absorption = (*S* − *W*)/*W* × 100,

where *W* is the initial weight of scaffold formulation (mg), and *S* is Weight of scaffold formulation (mg) after soaking in water until saturation (after 24 h). The porosity was calculated by the following equation.

Porosity = (*M*2 − *M*1)/*ρV* × 100

where *M*1 is the mass of the scaffold before immersion in absolute ethanol, *M*2 is the mass of the scaffold after immersion in absolute ethanol, *ρ* is the density of absolute ethanol, and *V* is the volume of the scaffold from geometry calculation.

### 3.6. Morphology

The morphological analysis of the composite scaffold was done using scanning electron microscopy (SEM). The scaffold was coated with gold using an Edwards E306 sputter coater. The stubs were introduced into the specimen chamber of a VEGA3 TESCAN scanning electron microscope. The stubs mounted on the stage could be tilted, rotated and moved to the desired position and orientation [44]. The scaffold was viewed under different magnifications in different sizes according to the nature of the fibers.

### 3.7. In-Vitro Drug Release Studies

The scaffold was attached to the Franz diffusion cell in such a way that GS was released into the receptor compartment, which was filled with (5 mL) any one of the dissolution mediums such as water, physiological fluid (PSF) and phosphate buffer saline (PBS) solution of pH 7.4 at 37 ± 1 °C. The elution medium was stirred magnetically, and the aliquots (1 mL) were withdrawn at predetermined time intervals up to 16 h and replaced with the same volume of fresh dissolution medium [45]. The collected samples were diluted with equal volumes of the medium, and the absorbance was recorded depending on the λmax of the medium (water, PSF and PBS) using a UV-spectrophotometer (Agilent Cary, NC, USA).

### 3.8. Biocompatibility Test

The monolayer of fibroblast cell line NIH 3T3 cell viability in terms of metabolic activity was determined calorimetrically by assaying the uptake of 3-(4, 5-dimethylthiazol-2-yl)-2, 5-diphenyl-tetrazolium bromide (MTT) by the cells. The formazan (metabolic) product of MTT was solubilised using acid isopropanol (0.04N HCl in isopropanol) and read on a spectrophotometer at 550 nm. MTT assay results were expressed as cell numbers using a standard curve constructed from cells seeded in 12 culture well plates. The passaged cells were seeded on the scaffold at the density from 8 × 10^3^ to 7 × 10^6^ cells/well and allowed to adhere for 6 h at 37 °C humidified with 5% CO_2_. The absorbance values were plotted against the exact cell numbers to establish a standard calibration curve for the calculation of cell numbers in the scaffold [46].

### 3.9. Statistical Analysis

The presented data values were determined in terms of mean ± standard deviation (SD). The difference in statistical significance values was expressed from Student’s *t*-test and one-way ANOVA analysis. A *p*-value of < 0.05 was measured as statistically significant.

## 4. Conclusions

The novelty of this work was that the scaffold established using human waste hair keratin biomaterial has been demonstrated to be a promising formulation in the field of soft tissue engineering, i.e., wound healing. The extracted hair keratin was capable of forming a scaffold with the cross-linking polymer of PVA. A controlled release of gentamicin sulphate from the developed scaffold (1:4) indicated its feasibility for drug delivery that promotes biomedical application by reducing bacterial infections. The MTT assay proved that prepared scaffolds are biocompatible, capable of producing better biocompatibility, and the prepared scaffolds are porous materials with a fibrous complex, have a suitable claim in the biomedical field for uses such as wound healing and drug delivery. Future research could investigate further characteristics of the scaffold, such as tensile strength and in vivo performance.

## Figures and Tables

**Table 1:** Swelling, water absorption and porosity of scaffold (Mean ± SD, *n* = 3).

Scaffold Ratio	Swelling Index (%)		Water Absorption (%)	Porosity (%)
	Size	Weight		
1:3	1.52 ± 0.15	26 ± 2.31	72.93 ± 9.26	68.93 ± 1.33
1:4	2.04 ± 0.09	31 ± 2.88	83.64 ± 14.29	66.05 ± 1.97

**Table 2:** Formulation of scaffold.

Ratio	mg/50 mL (*w/v*)			
	Keratin	PVA (2%)	Alginate Dialdehyde	GS
1:2	4	8	5	4
1:3	4	12	5	4
1:4	4	16	5	4

The quantities mentioned are in mg for 50 mL of the scaffold formulation.

## Data Availability

Data is contained within the article.
